# Utilization of Methionine Sources for Growth and Met+Cys Deposition in Broilers

**DOI:** 10.3390/ani10122240

**Published:** 2020-11-29

**Authors:** Andreas Lemme, Victor Naranjo, Juliano Cesar de Paula Dorigam

**Affiliations:** Evonik Operations GmbH, Rodenbacher Chaussee 4, 63457 Hanau, Germany; victor.naranjo@evonik.com (V.N.); juliano.dorigam@evonik.com (J.C.d.P.D.)

**Keywords:** broiler, relative bioavailability, methionine, methionine hydroxy analogue, utilization

## Abstract

**Simple Summary:**

Methionine (Met) is the first limiting amino acid in broiler feeds and for balancing dietary Met and methionine+cysteine (Met+Cys) levels. DL-2-hydroxy-4-methylthio butanoic acid (HMTBA) and DL-methionine (DLM) are typical feed additives. The relative bioavailability value (RBV) describes the nutritional value of HMTBA relative to DLM and is important for adequate, precise, and cost-effective broiler nutrition. The current broiler feeding trial revealed an average RBV of 63% compared to DLM and the inclusion of an internal standard into the experimental design allowed for validation of the methodological approach. Evaluation of the utilization of supplemental Met sources for Met+Cys deposition in body protein provided further evidence for a higher efficiency and, thus, nutritional value of DLM over HMTBA.

**Abstract:**

Knowledge about the nutritional value of methionine sources is highly important for their appropriate application in terms of animal and economic performance. Therefore, a broiler feeding trial was conducted to determine the relative bioavailability value (RBV) of DL-2-hydroxy-4-methylthio butanoic acid (HMTBA) compared to DL-methionine (DLM). DLM diluted to 65% purity (DLM65) served as the internal standard, with a known RBV of 65%. A total of 1920 d-old male broilers were used in the three-phase feeding trial comprising 16 treatments including a basal, Met+Cys-deficient diet and 5 graded DLM, HMTBA, or DLM65 levels. Growth performance and carcass quality data were subjected to multi-exponential regression analysis. Increasing levels of any Met source significantly improved all performance parameters compared to the negative control (*p* < 0.05). Across all performance parameters, the RBV of HMTBA was 63% and that of DLM65 was 58%. All RBV estimates of HMTBA and DLM65 were significantly lower than 88% (*p* < 0.05). Cumulative efficiency of DLM for Met+Cys deposition in body protein was higher than that of HMBTA at any dose, confirming the determined RBV. Using DLM65 as an internal marker allowed for validation of the methodology.

## 1. Introduction

Supplemental methionine (Met) sources are commonly used to precisely meet the sulphur amino acid requirements of broilers. The global production capacity for supplemental Met sources is estimated to be >1 million tons, from which the majority is supplied as dry DL-methionine (DLM, 99% content) followed by liquid DL-2-hydroxy-4-methylthio butanoic acid (HMTBA, 88% content) [1]. While both products provide Met activity to birds, there is an ongoing discussion about the nutritional value of HMTBA compared to DLM as from the chemical point of view, HMTBA is not an amino acid due to the replacement of the characteristic amino group (-NH2) by a hydroxy group (-OH) [2]. The nutritional value expressed as relative bioavailability value (RBV) of HMTBA compared to DLM, which also indicates the replacement ratio of these products in the feed, is a critical factor for cost-effective purchasing and optimization of feed costs in the feed manufacturing business [3].

Various publications and literature reviews indicated an RBV of about 65% for HMTBA compared to DLM in broilers on a product-to-product basis [3,4,5,6]. However, the experimental setup to determine the RBV of these Met sources has been a matter of recent discussion [7] leading to controversial RBV estimates. While Vázquez-Añón et al. [8] claimed that the two methionine sources would result in different shapes of response to be analyzed by different regression models, the same authors suggested multiple regression approaches with the same quadratic shape for both methionine sources in a second paper in the same year [7]. Moreover, Kratzer and Littell [9] suggested that the maximum performance achieved with the methionine sources would be different. While this was doubted in a quick reply by Piepho [10], a meta-analysis provided statistical evidence that both methionine sources would allow for the same maximal performance (asymptote), while the steepness of the curve indicates their nutritional value [3]. Earlier experiments [11,12,13,14] provided additional evidence on the appropriateness of the simultaneous dose-response approach followed by multi-exponential regression to determine RBV by introducing diluted DLM as an internal standard. The principle behind the RBV determination is describing the biological response to graded supplementation levels of each tested product by either linear functions (slope ratio) or simultaneous exponential functions [15]. In this approach, the starting point (basal diet) of the curve and the maximum response asymptote are the same for both products. Therefore, relating the regression coefficient of the DLM and HMTBA equations reveals the RBV, as the only difference between the curves will be their steepness. The method of simultaneous exponential regression has been used extensively to compare other nutrient sources such as phosphorus [16,17,18], iron [19], manganese [20], and lysine [21]. In addition, a recommended RBV of 65% for HMTBA relative to DLM has been challenged by a number of experiments in which this recommendation has been applied. For instance, Payne et al. [22], Goes et al. [23], and Murakami et al. [24] reported dose–response trials in which DLM was supplemented as high as 65% of the respective HMTBA doses. Results confirmed that broiler performance did not differ at each of the corresponding pairs of treatment, while multi-exponential regression confirmed an RBV of about 65%. In addition, these trials suggest that the recommendation of RBV of 65% for HMTBA compared to DLM is applicable independent of the targeted performance and Met+Cys level.

Still, the experimental setup has been a matter of discussion. Liu et al. [25] and Agostini et al. [26] tested HMTBA against DLM in a dose-response approach but dosing the methionine sources only slightly below, at, and above the requirement and could not find differences between the products. Ullrich et al. [27] and Ribeiro et al. [28] tested various methionine sources only at one or two supplementation levels, which were at or above the requirement and did not observe differences between treatments. While suggesting testing products at requirement levels, researchers proposed linear models on the utilization of methionine sources for methionine deposition in the animal and concluded better utilization of HMTBA compared to DLM [25,26]. Therefore, a trial was conducted to determine the RBV of HMTBA compared to DLM for common performance parameters and to validate the suitability of the multi-exponential regression analysis for estimating the RBV by using diluted DLM (DLM65) as an internal standard. Furthermore, responses to the methionine sources were used to determine the efficiency of utilization of methionine sources for Met+Cys deposition.

## 2. Materials and Methods

The experiment was conducted according to the guidelines of the Animal and Human Welfare Codes/Laboratory practice codes in the Netherlands. The protocol was approved by the Ethics Review Committee under project number AVD246002016450. The experiment was conducted in the facilities of Schothorst Feed Research, 8200 AM Lelystad, The Netherlands.

A total of 1920 day-old male Ross 308 (average BW: 39.1 ± 0.46 g) were allocated to 96 floor pens of 20 broilers each. Each pen (2 m^2^) had one feeder, a line of drinking nipples, and wood shavings as bedding material. Ambient temperature was gradually decreased from 34 °C at arrival of chicks to 20 °C at 35 days of age. Light was continuously on for the first 24 h to give birds the opportunity to readily find feed and water. After that, the light schedule was 22L:2D during one day, and then changed to 8L:4D:10L:2D during the remaining experimental period, complying with the EU legislation of a minimum of six hours of darkness from the second day onwards [29].

Birds and feed were weighed at day 0 (placement) and at the end of each feeding phase at days 11, 28, and 35, allowing for calculation of body weight gain (BWG), feed consumption (FI), and feed conversion ratio (FCR). On day 35, two birds per pen, which were close to the average BW of the pen, were selected for carcass evaluation and individually weighed. The carcasses were chilled for 4 h before being cut up. Carcass weight was defined as plucked, bled, and eviscerated carcasses without head, neck, and feet. Carcass yield (CY) was expressed as percentage of BW. Breast meat weight included both musculus pectoralis major and musculus pectoralis minor (without skin). Breast meat yield (BMY) was expressed as percentage of BW.

Diets consisting of corn, wheat, and soybean meal as the main ingredients were formulated to meet or exceed the ideal AA profile [30], except for digestible Met and Met+Cys (Table 1). In order to increase the probability of achieving significant responses to supplemental Met sources as well as to produce a complete response curve, dietary digestible Met+Cys was minimized in the basal diets. Accordingly, digestible Met+Cys levels of 6.2, 5.4, and 5.1 g/kg were targeted in the basal starter, grower, and finisher diets, respectively, meeting recommended levels by about 68% in each phase. Prior to feed manufacturing, main ingredients were analyzed for crude protein (CP) and total amino acids by near infrared spectroscopy [31,32] and results were used for diet formulation. A 3-phase feeding schedule included starter (d 0 to 11), grower (d 11 to 28), and finisher (d 28 to 35) diets. Each phase comprised 16 treatments (6 replicate pens per treatment, 20 birds per pen) including a basal diet deficient in digestible Met+Cys without supplemental Met sources, and 3 × 5 diets with graded levels of either DLM (MetAMINO^®^, Evonik Operations GmbH, Essen, Germany; min. 99% DL-Methionine), HMTBA (Rhodimet AT88^®^, Adisseo France S.A.S, Antony, France, min. 88% DL-2-hydroxy-4-methylthio butanoic acid), or DLM65 (including MetAMINO^®^, Evonik Operations GmbH, Germany) (Table 2). Starch was used to dilute DLM to a Met content of 65% in DLM65. Met sources were always added on a weight-to-weight basis at 0.40, 0.80, 1.20, 2.10, and 3.00 g/kg. Starter feeds were produced in crumbles, while grower and finisher diets (3.0 mm) were steam pelleted. Salinomycin was added to the starter and grower feeds as an anticoccidial agent, while no coccidiostat was added to the finisher diets. Feed and water were supplied ad libitum throughout the experimental period.

Raw data were evaluated for outliers per measurement period and significant outliers (outside the range of mean ± 2.5 times SD) were excluded from statistical analysis. If at least one of the response parameters FI, BWG, or FCR was identified as an outlier, the entire record for that experimental unit was excluded. Accordingly, one replicate of treatment 7 was excluded. The experimental data were analyzed by ANOVA using Genstat for Windows (17th version). Treatment means were compared by least significant difference, as shown in the following model:

Y_jk_ = µ + Block_j_ + Treatment_k_ + e_jk_ (1)

where Y_jk_ is the dependent variable, µ is the overall mean, Block_j_ is the block effect (j = 1…6), Treatment_k_ is the effect of treatment (k = 1…16), and e_jk_ is the residual error.

For the RBV determination of HMTBA and DLM65 compared to DLM, data were analyzed by simultaneous multi-exponential function [15] using Genstat for Windows (17th version):

Y = B1 + B2 × (1 − e^(B31 × X1 + B32 × X2 + B33 × X3)^) (2)

where X1 is the level of DLM, X2 is the level of HMTBA, X3 is the level of DLM65 (all products in g/kg), B1 is the intercept, B1 + B2 is the asymptote, B31 is the steepness coefficient for DLM, B32 is the steepness coefficient for HMTBA, and B33 is the steepness coefficient for DLM65. Ratio of B32/B31 indicates the bioavailability values for HMTBA relative to DLM and B33/B31 indicates the bioavailability values for DLM65 relative to DLM. In addition, the null hypothesis whether asymptotes for the products DLM, HMTBA, and DLM65 would differ was tested.

Data are presented as means. Values with *p* ≤ 0.05 were considered statistically significant, whereas 0.05 < *p* < 0.10 was considered a trend.

Met+Cys deposition estimates were used for the determination of the marginal and cumulative efficiency of Met sources. To estimate the deposition of Met and Cys in body protein, concentrations of these amino acids in broiler body protein are required. As no whole body analyses were included in this study, average concentrations of 3.5 g/kg Met, 2.8 g/kg Cys, and 6.3 g/kg Met+Cys in body weight were assumed, respectively. These concentrations were obtained from Khan [33], who examined amino acid composition in the whole body of various broiler genetics at different ages. Average body weights in the current study ranged between 1532 and 2590 g (Table 3) and according to Khan [33], Met, Cys, and Met+Cys levels in whole body protein would not vary any more in this weight range. It is recognized that the impact of Met-source supplementation affected growth performance and allometric growth, as evidenced by effects on breast meat yield which might influence the Met, Cys, or Met+Cys deposition. However, this impact was considered minor for the purpose of this examination. For this exercise, DLM and DLM65 treatments were merged. Product intakes were revealed by multiplying feed intake with supplementation levels. Analyses were based on replicates with n = 35 for HMTBA and n = 66 for DLM+DLM65. The determination of efficiency followed the 4-parameter logistic model [34,35]:

Y = (Y_max_ + [b × (1 + c) − Y_max_] × e^kx^)/(1 + c × e^kx^) (3)

where X is dietary Met source intake (mg/bird), Y is Met+Cys deposition (Body weight, mg/bird), Y_max_ is asymptotic maximum response at infinite intake, b is Y-intercept or response to zero intake, c is the parameter related to the inflection point or shape, and k is the parameter related to the scale of the data.

While cumulative efficiency was determined by relating the Met+Cys deposition above basal treatment against product intake, marginal efficiency was using 1 derivation of the above 4-parameter logistic equation:

ΔY/ΔX = (k × e^−kx^)/(1 + c × e^−kx^) × (Y_max_ − b) × (1 − c) (4)

## 3. Results and Discussion

Analyzed values of amino acids in the experimental diets from all phases were in close agreement with the calculated values (Table 1). Recovery rates ranged from 97 to 109% for total Met+Cys and Lys. The high accuracy in the analytical results was accomplished by formulating diets based on the analyzed values of the main ingredients and manufacturing feeds in a specialized research feed mill with confirmed dosing accuracy and mixing quality. Therefore, the calculated levels of Met product additions were used for response evaluations.

### 3.1. Performance and Relative Bioavailability Determination

Increasing levels of either DLM, HMTBA, or DLM65 improved growth performance (Table 3) and carcass yields (Table 4) compared to the basal diet. Results are reported in absolute values but also relative to the basal treatment. Accordingly, the highest addition, 3.00 g/kg, significantly improved BWG by 70, 67, and 70% (*p* < 0.05) and reduced FCR by 22, 22, and 23% (*p* < 0.05) for DLM, HMTBA, and DLM65, respectively. Moreover, birds of these treatments achieved 5–7% higher BW than suggested by the broiler breeder [36] at the highest supplementation level, implying that performance was not limited by any other factor than Met+Cys supply in this trial. Similarly, CY was improved by 13, 11, and 13% (*p* < 0.05) and BMY by 62, 60, and 63% (*p* < 0.05) for DLM, HMTBA, and DLM65, respectively. Moreover, responses reported in Table 3 and Table 4 showed gradual significant improvements with increasing supplementation of either Met source (*p* < 0.05). These relative responses confirm that the main pre-requisites for an appropriate RBV determination were successfully met such as: (1) a common starting point (basal) where dietary Met+Cys was clearly deficient, (2) three or more supplementation levels of each test product were included in the study, and (3) graded supplementation of the products resulted in graded dose–responses [4]. The latter point is important as without response or responses only nearby asymptotic performance, misinterpretations of RBV are likely to occur. For example, if only the basal treatment and the 3rd, 4th, and 5th inclusion level of the DLM, DLM65, and HMTBA would have been considered in the current trial, RBV determination would have likely revealed the same efficiency for all Met sources. It is difficult to believe that DLM65 with 65% purity is equally effective as DLM with 99%; and, in fact, including DLM65 as an internal standard with a priori known RBV illustrates the risk for misinterpretation of data but also for inadequate application of the RBV model. For example, Agostini et al. [26] and Ullrich et al. [27] drew such conclusions when they supplemented Met sources close to and above requirements. A further requirement for RBV determination by multi-exponential regression is that responses are non-linear as otherwise, a slope-ratio would be applicable [15]. Response data expressed on a relative scale in Table 3 and Table 4 clearly confirm non-linear responses. Moreover, within the performance criterion, these relative but also absolute numbers suggest common asymptotes and, therefore, confirm findings by Sauer et al. [3]. Statistical analysis also provided evidence that asymptotes achieved with the products did not differ (null hypothesis retained), confirming the outcome of earlier research [3,10].

**Figure 1:**
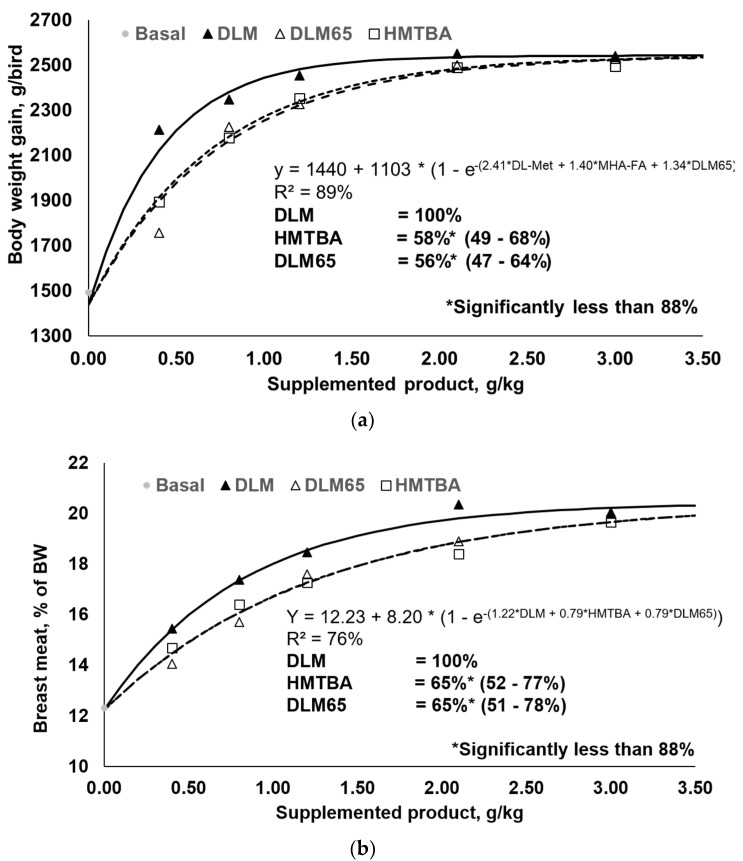
Body weight gain (**a**) and breast meat yield (% of body weight) responses (**b**) of 0–35 day old broilers to graded levels of DL-2-hydroxy-4-methylthio butanoic acid (HMTBA), or to 65% purity diluted DLM (DLM65) relative to DL-Methionine (DLM).

One objective of this study was to determine the RBV of HMTBA and DLM65 relative to DLM and, as outlined above, all respective requirements were met. Responses to increasing levels of DLM, HMTBA, and DLM65 and respective regression analyses are shown in Table 5 as well as in Figure 1 (only body weight gain and breast meat yield plotted) for performance criteria. Multi-exponential regression analysis revealed HMTBA to be 58% and 66% as efficacious as DLM for BWG and FCR, respectively. Similarly, the estimated RBV of DLM65 were 56% and 54% for BWG and FCR. Based on carcass traits, HMTBA was estimated to be 63% and 65% as efficacious as DLM for CY and BMY. Correspondingly, the estimated RBV of DLM65 were 58% and 65% for these parameters. As summarized in Table 5, all RBV estimates were significantly lower than 88% (*p* < 0.05) as this is the basis for comparison. HMTBA contains a minimum of 88% methionine hydroxy analogue, while the remainder is water and impurities. Met sources were supplemented on a product basis and, therefore, an RBV of maximal 88% could be expected. Based on all the evaluated parameters, the average RBV of HMTBA and DLM65 compared to DLM was 63% and 58%, respectively. The average RBV of 58% for DLM65 was close to the expected value of 65% (because of dilution to 65% purity), providing evidence for the appropriateness of using a simultaneous dose–response approach to determine the RBV of nutrient sources. Indeed, there was a difference of 7% points, but the results of this study fit well to earlier findings [11,12,13] (Table 6). While there was little variation between experiments, DLM65 was 63% as efficient as DLM on average across six experiments, whilst HMTBA was 62% as efficient. This compilation ultimately validates the methodology because RBV of DLM65 almost exactly met the expectation for DLM diluted to 65% purity. Moreover, HMTBA showed similar efficiency which, in any case, was significantly lower than DLM (*p* < 0.05).

With validation of the method by DLM65, consequently, the determined RBV for HMTBA would also be validated. The average RBV of HMTBA compared to DLM was 63%, in line with earlier meta-analyses [3,4]. Agostini et al. [26] using the same regression model could not find significant differences between slopes for HMTBA and DLM curves (weight gain, FCR), concluding that there are no differences in RBV between products. In addition to the earlier mentioned shortcomings of that study, and in contrast to suggestions by Littell et al. [15] and Sauer et al. [3], a common asymptote for both products was not assumed, and the first inclusion level of both products allowed almost maximum performance. This means that the curvilinear part of the response curve was not well described and defined by the data. Similar conditions can be reported for other studies [25,27,28] (Ullrich et al., no dose response). With the findings on DLM65 in mind, we conclude that such trial setups are not suitable for the determination of biological efficiency between amino acid sources and that respective conclusions from such experiments are misleading.

The lower RBV of HMTBA might be caused by reduced intestinal absorption due to microbial degradation [37,38,39] or poor absorption of the di- and oligomers of HMTBA especially [37,40,41,42], inefficient conversion of HMTBA to L-Met after absorption [43], or a combination of both, among others. Studies by Maenz and Engele-Schaan [37], Maenz and Engele-Schaan [41], and Lingens and Molnar [42] using radiolabeled Met sources indicated a significantly lower absorption of HMTBA compared with DLM in broilers. Research by Lemme and Mitchell [40] confirmed this, but additional findings demonstrated that particularly the absorption of oligomers of HMTBA is lower than the absorption of DLM, as previously suggested [44]. This is in contradiction to other findings observing hydrolyzation of HMTBA oligomers into monomers [45]. Moreover, it has been demonstrated that intestinal absorption is not a limiting point for HMTBA, since it appears to be completed by the end of the duodenum in chickens [46]. Another potential explanation of the lower RBV of HMTBA could be linked to the differences in metabolic rate between sources, leading to higher trans-sulfuration of HMTBA compared to DLM, resulting in higher Cys and taurine concentrations [45]. Drew et al. [39] and Malik et al. [38] reported that a significant portion of monomeric HMTBA is degraded by intestinal microbes in poultry and swine and, therefore, is not available to the host anymore—probably explaining most of the lower RBV of HMTBA.

### 3.2. Utilization of Methionine Sources for Met+Cys Deposition in Body Protein

**Figure 2:**
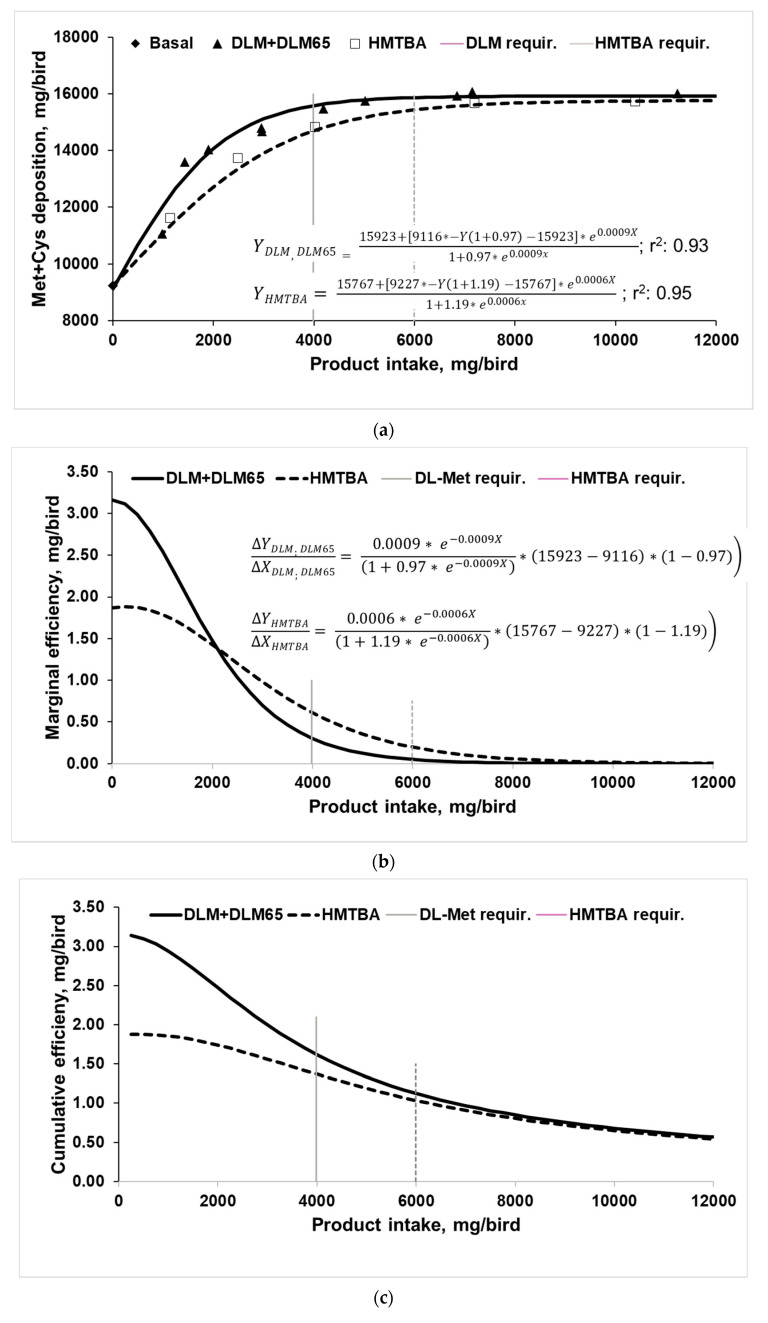
(**a**) Methionine + Cysteine (Met+Cys) deposition of 0–35 day old broilers fed increasing levels of DL-Methionine (treatments DLM and DLM65) and DL-2-hydroxy-4-methylthio butanoic acid (HMTBA) (A; four-parameter logistic regression analyses on replicate basis, treatment means are plotted in the diagram, required product intake for 95% of asymptotic response 3984 mg DLM and 5991 mg HMTBA); (**b**) marginal efficiency of ingested products for Met+Cys deposition according to the 1st derivation of the four-parameter logistic regression equation; (**c**) cumulative efficiency of ingested products for Met+Cys deposition.

In Figure 2, Met+Cys deposition (a, marginal (b), and cumulated utilization (c) of Met sources are shown in relation to product intake. Accordingly, growth responses were used to estimate the Met+Cys deposition, which means responses above the negative control. This was basically the approach also used by Vázquez-Añón et al. [8], Liu et al. [25], and Agostini et al. [26] as it suggests that any response above control treatment is attributable to the addition of Met sources. However, while the mentioned authors referred to body weight gain responses, we estimated the Met+Cys deposition above control. Body composition analyses to directly determine Met+Cys deposition were not performed in this experiment, but 0.63% Met+Cys in body weight was assumed [33]. The fate of the ingested Met sources for either Met or Cys deposition cannot be distinguished. This is especially an issue in vegetable-based diets as fed in the current trial because those are usually co-limiting in Cys, assuming an optimal dietary Met to Cys ratio of 52:48 [47,48]. Therefore, efficiency for Met+Cys deposition rather than only for Met deposition was chosen as a response parameter because Met sources were likely incorporated as Met as well as Cys. This is in contrast to Fatufe and Rodehutscord [34], who related their analysis to Met deposition only. For that study also, it cannot be excluded that a certain amount of supplemented Met was transformed to and deposited as Cys. In any case, the same mathematical approach (four-parameter logistic regression) was applied for the current dataset. While Fatufe and Rodehutscord [34] investigated the utilization of total Met (protein-bound + free) from a very deficient supply situation up to the asymptotic level, the current study focused only on the utilization of Met-sources at Met levels above that supplied with the basal diet.

The basal diet allowed for deposition of about 9200 mg Met+Cys/bird, whereas increasing intake increased Met+Cys deposition to about 15,800 to 15,900 mg/bird (Figure 2a). Both regressions describe essentially the same asymptote—despite being done separately—and curves differ only in shape. Calculation of the required product intake to achieve 95% asymptotic response revealed 3984 mg DLM/bird and 5991 mg HMTBA/bird, indicating a higher requirement for HMTBA. Moreover, only 66.5% of HMTBA intake was needed with DLM to achieve 95% asymptotic response. This basically confirms earlier multi-exponential regression analyses for various performance criteria (Table 5).

The first derivation of the four-parameter logistic regression equation provides information on the marginal efficiency of the supplemented Met sources for Met+Cys deposition (Figure 2b, [34,35]). Accordingly, marginal efficiency reduced with increasing intake and approached zero, when Met+Cys deposition (Figure 2a) achieved the asymptotic level. Thus, the first unit of either Met source was utilized best for Met+Cys deposition and followed then, the law of diminishing returns. The DLM curve begins at 3.1 mg Met+Cys deposition per mg product addition, while the HMTBA curve begins at 1.9 mg Met+Cys deposition, suggesting a higher marginal utilization of DLM. Interestingly, in this part of the curve, more Met+Cys was gained than added with the products. An explanation for this finding would be that increasing levels of either Met source increased feed intake, especially at lower supplementation levels (Table 3), which consequently resulted in higher intakes of protein-bound Met and Cys on top of the supplemented methionine. Met deficiency, which was provoked with the basal diet, represents an amino acid imbalance which usually results in a depression in feed intake [49]. Increasing the supply of the respective amino acid will alleviate this effect. A further mechanism might have played a role too. It assumes that part of the increased dietary Met is transformed into Cys because this is a well-established way to meet relative Cys deficiency [50]. While it is not possible to exactly follow the routes of Cys and Met for deposition with the current experimental approach, increasing levels of dietary Met might have allowed for making unused Cys available. According to Liebig’s law of the minimum and the concept of the well-known Liebig barrel, Met might have been the first performance-limiting amino acid, while Cys was the second limiting. According to Verstegen and Jongbloed [51], any relative surplus of amino acids cannot be stored but will be degraded and the nitrogen will be excreted. Increasing supplementation with dietary Met would reduce the relative surplus of many (essential) amino acids, but also Cys. Consequently, the relative surplus of Cys compared to Met was degraded. However, incremental supplementation of Met first covered the gap for Met, while the relative surplus of Cys gradually decreased to zero, as it could be utilized for deposition. Only after that, supplemental Met served as both the Met source and Cys precursor. While the HMTBA started at a lower efficiency, the curve is flatter than that of DLM and at about 2000 mg product intake curves crossed each other (Figure 2b). The latter is explained by the fact that DLM became closer towards the asymptotic level for deposition. Agostini et al. [26] also presented marginal efficiency data (mg extra Met/g extra body weight gain) for Met sources and although they used a linear model, the regression lines for DLM and HMTBA crossed each other both in male and female broilers. When expressing their data in a reciprocal, inverse way (g extra body weight/mg extra Met), the plots show similarities to those in Figure 2b (product intake < 2000 mg/bird) as at lower dietary doses, more body weight was gained per mg of DLM and less per mg of HMTBA. At higher dietary supply, this changed to the opposite because also in the trial by Agostini et al. [26], DLM-fed birds achieved the asymptote earlier than the HMTBA fed broilers. The authors deliberately set the supplementation levels close to and above the requirement level, which means close to the asymptotic level. This would be represented with >2000 mg product intake in the current trial. These relatively high supplementation levels and the rather small range let the marginal utilization response appear linear rather than non-linear as observed in our study, which covered a broader range of dietary Met source supply. In any case, at marginal Met supply, DLM was more efficient than HMTBA in the Agostini et al. [26] experiment as well. Similarly, Liu et al. [25] plotted body weight gain above basal treatment against Met intake above basal (equaling intake of HMTBA and DLM) and analyzed the responses by linear regression. While their plot suggested a data cloud rather than linear trends, their slope-ratio analysis suggested a 20% higher slope for HMTBA than for DLM. Indeed, the slope represents the marginal efficiency of Met sources for body weight gain but would again support our findings at >2000 mg intake. The first inclusion level resulted in performance close to asymptote, while with the 2nd and 3rd inclusion levels, the maximum was already achieved [25]. Therefore, the proposed higher availability for HMTBA is due to the law of diminishing returns and would in fact suggest a lower cumulative efficiency. Vázquez-Añón et al. [8] analyzed four broiler dose–response datasets in a similar way to Liu et al. [25] and found only partially linear relationships but also quadratic, exponential, and broken-line responses. Although they concluded that birds responded differently to Met sources within and between trials, the regression lines of the different models crossed, suggesting higher marginal efficiency for DLM at lower and higher marginal efficiency for HMTBA at higher product inclusion levels. Moreover, Vázquez-Añón et al. [8] analyzed combined data of all four trials and concluded that increasing HMTBA supplementation linearly increased weight gain above control, while the response to DLM was of quadratic nature. Indeed, this data compilation as well as each single trial exactly confirms the above conclusion that marginal utilization declines once maximum performance has been achieved. Having commented on their overall conclusion [8], it should be mentioned that the magnitude of responses (above control) differed greatly between trials from about 100 g (trial 4) up to 470 g (trial 2), which would strongly impact the marginal utilization per se but also make a combination of data in this way questionable.

Fatufe and Rodehutscord [34] and Fatufe et al. [35] concluded that at the point of maximal marginal Met or Lys utilization, broilers achieved only 40–50% of maximal Met or Lys deposition. In contrast, marginal Met or Lys efficiency was only 20% at maximal Met or Lys deposition (95% of asymptotic response). This pattern is clearly reflected in the current trial, as utilization of Met sources was highest at lower supply where Met+Cys deposition was still low, while marginal Met+Cys efficiency approached zero when achieving maximal deposition.

Marginal efficiency of zero at high product intake does not mean that cumulated utilization of total dietary Met+Cys intake nor that of total Met source intake approaches zero because it describes the utilization of just the next unit of product intake. When evaluating the cumulated efficiency of Met product intakes (Figure 2c), response curves did not achieve zero efficiency but continued to decrease until the highest intake. Basically, if maximum Met+Cys deposition is achieved but intake still increases due to further product supplementation, cumulative utilization shrinks. Cumulative HMTBA efficiency curve does not cross any more the DLM curve, but curves came to a similar efficiency as soon as maximal Met+Cys deposition was achieved with both products at about 8000 mg/bird intake. This ultimately leads to the conclusion that overall ingested DLM was utilized more efficiently than HMTBA at any supplementation level until maximal depositions were achieved. Fatufe and Rodehutscord [34] reported a similar observation, as marginal efficiency response curves to methionine supply at two dietary protein levels crossed, while the cumulative efficiency curves approximated each other but—at least in the tested range of dietary Met intake—did not meet.

## 4. Conclusions

These results demonstrate that the RBV of HMTBA is significantly lower than its active content of 88% and close to 65% in agreement with previous publications. The inclusion of diluted DLM65 confirmed that the multi-exponential regression analysis is a valid approach to estimate the bioavailability of Met sources and resulted in a similar RBV (61 vs. 65%) as liquid HMTBA. In terms of cumulative utilization, DLM outperformed HMTBA at any supplementation level, until maximal Met+Cys deposition was achieved with 5991 mg HMTBA/bird. At this level, curves of both products converged.

## Figures and Tables

**Table 1:** Ingredient and nutrient composition (g/kg) of the basal diets.

Ingredients	Starter (day 0 to 11)	Grower (day 11 to 28)	Finisher (day 28 to 35)
Corn	250.0	208.8	200.0
Soybean meal	323.7	255.8	218.6
Wheat	286.1	364.6	365.9
Peas	50.0	100.0	139.4
Corn gluten meal	18.2	0.0	0.0
Soybean oil	14.4	11.3	17.3
Lard	19.4	28.6	34.1
Limestone	14.5	11.6	9.0
Monocalcium phosphate	7.6	4.3	1.3
Salt	3.3	2.5	2.6
Sodium bicarbonate	0.0	1.0	1.0
L-Lysine-HCl	2.7	1.8	1.4
L-Threonine	1.1	0.9	0.7
L-Valine	0.7	0.5	0.3
Phytase	3.3	3.3	3.3
Premix ^1^ + coccidiostat ^2^ + NSP degrading enzyme	5.0	5.0	5.0
**Nutrient composition**			
AMEn (kcal/kg)	2900	2975	3075
Ash	5.55	4.62	3.94
Crude protein	233.5	205.2	194.2
Crude fibre	23.4	24.4	25.1
Starch (AM)	349.4	385.8	396.5
Crude fat (AH)	60.0	64.5	75.6
Calcium	7.86	6.11	4.61
Phosphorus	5.39	4.47	3.72
Sodium	1.45	1.40	1.40
Chloride	3.15	2.49	2.42
Potassium	9.85	8.99	8.53
DEB (mEq/kg)	226	221	211
**Amino acids**			
Total Lysine-calculated	14.1	12.0	11.2
Total Lysine-analyzed ^3^	14.5 (103%)	12.5 (104%)	11.7 (104%)
Total Met + Cys–calculated	7.1	6.2	5.8
Total Met + Cys–analyzed ^4^	7.4 (101%)	6.2 (100%)	6.3 (108%)
Dig. Lysine ^5^	12.6	10.7	9.90
Dig. Methionine+Cysteine	6.2	5.4	5.1
**Dig. AA: Dig Lysine**			
Methionine	24	24	24
Methionine+Cysteine	49	50	51
Threonine	65	65	65
Tryptophan	20	21	21
Arginine	106	111	114
Isoleucine	68	69	70
Valine	79	80	80
Leucine	132	127	129
Glycine+Serine	138	143	145
Glycine_equivalents_^6^	115	120	122

^1^ per kg of feed: Vitamin A 10,000 IE, Vitamin D3 2500 IE, Vitamin E 50 mg, Vitamin K3 1.5 mg, Vitamin B1 2.0 mg, Vitamin B2 7.5 mg, Vitamin B6 3.5 mg, Vitamin B12 20 μg, Niacin 35 mg, D-pantothenic acid 12 mg, Choline chloride 460 mg, Folate 1.0 mg, Biotin 0.2 mg, Iron 80 mg (265 mg FeSO_4_ H_2_O), Copper 12 mg (48 mg CuSO_4_ 5H_2_O), Manganese 85 mg (140 mg MnO), Zinc 60 mg (165 mg ZnSO_4_ H_2_O), Iodine 0.8 mg (1.2 mg KJ), Selenium 0.15 mg (0.33 mg Na_2_SeO_3_). ^2^ Salinomycin, only in starter and grower diets. ^3^ Across all 16 experimental diets; in brackets: relative to calculated. ^4^ Basal treatment (in brackets: relative to calculated). ^5^ Digestible amino acids are based on apparent fecal digestibility. ^6^ Glycine equivalents = Glycine + 0.714 × Serine.

**Table 2:** Experimental design and supplementation levels of DL-methionine (DLM), DL-2-hydroxy-4-methylthio butanoic acid (HMTBA), and to 65% purity diluted DLM (DLM65).

Treatment	Methionine Sources	Addition of Methionine Sources (g/kg) ^1^			Replicates per Treatment	Birds per Treatment
		0–11 day	11–28 day	28–35 day		
1	Negative control (NC)	0.00	0.00	0.00	6	120
2	DLM	0.40	0.40	0.40	6	120
3	DLM	0.80	0.80	0.80	6	120
4	DLM	1.20	1.20	1.20	6	120
5	DLM	2.10	2.10	2.10	6	120
6	DLM	3.00	3.00	3.00	6	120
7	HMTBA	0.40	0.40	0.40	6	120
8	HMTBA	0.80	0.80	0.80	6	120
9	HMTBA	1.20	1.20	1.20	6	120
10	HMTBA	2.10	2.10	2.10	6	120
11	HMTBA	3.00	3.00	3.00	6	120
12	DLM65	0.40	0.40	0.40	6	120
13	DLM65	0.80	0.80	0.80	6	120
14	DLM65	1.20	1.20	1.20	6	120
15	DLM65	2.10	2.10	2.10	6	120
16	DLM65	3.00	3.00	3.00	6	120

^1^ Addition on product basis.

**Table 3:** Effect of increasing levels of Met sources on body weight gain, feed intake, and feed conversion ratio in broilers from 0 to 35 days of age.

Treatment	Met Source	Added Product	Body Weight Gain		Feed Intake		Feed Conversion Ratio	
		g/kg	g/Bird ^1^	Rel. ^2^	g/Bird ^1^	Rel. ^2^	g/g ^1^	Rel. ^2^
1	Basal	0.00	1492 ^a^	100	2809 ^a^	100	1.885 ^h^	100
2	DLM	0.40	2212 ^d^	148	3550 ^de^	126	1.605 ^e^	85
3	DLM	0.80	2347 ^e^	157	3649 ^efg^	130	1.555 ^cd^	83
4	DLM	1.20	2455 ^f^	165	3750 ^g^	134	1.528 ^bc^	81
5	DLM	2.10	2551 ^f^	171	3716 ^fg^	132	1.457 ^a^	77
6	DLM	3.00	2542 ^f^	170	3726 ^fg^	133	1.467 ^a^	78
7	HMTBA	0.40	1896 ^c^	127	3316 ^bc^	118	1.750 ^f^	93
8	HMTBA	0.80	2179 ^d^	146	3438 ^cd^	122	1.578 ^de^	84
9	HMTBA	1.20	2354 ^e^	158	3658 ^efg^	130	1.554 ^cd^	82
10	HMTBA	2.10	2489 ^f^	167	3709 ^fg^	132	1.490 ^ab^	79
11	HMTBA	3.00	2496 ^f^	167	3673 ^efg^	131	1.472 ^a^	78
12	DLM65	0.40	1756 ^b^	118	3181 ^b^	113	1.813 ^g^	96
13	DLM65	0.80	2226 ^d^	149	3592 ^ef^	128	1.614 ^e^	86
14	DLM65	1.20	2328 ^e^	156	3708 ^fg^	132	1.593 ^de^	85
15	DLM65	2.10	2501 ^f^	168	3726 ^fg^	133	1.491 ^ab^	79
16	DLM65	3.00	2529 ^f^	170	3688 ^fg^	131	1.459 ^a^	77
*p*-value			<0.001		<0.001		<0.001	

^1 a–h^ Values without a common superscript within a column differ significantly (*p* ≤ 0.05); ^2^ Rel: Relative response of each dietary treatment compared to the basal diet, which was set as 100%.

**Table 4:** Effect of increasing levels of Met sources on carcass yield and breast meat yield expressed as percentage of body weight at 35 days of age.

Treatment	Met Source	Added Product	Carcass Yield		Breast Meat Yield	
		g/kg	% of BW ^1^	Rel. ^2^	% of BW ^1^	Rel. ^2^
1	Basal	0.00	58.1 ^a^	100	12.3 ^a^	100
2	DLM	0.40	62.1 ^c^	107	15.4 ^cd^	125
3	DLM	0.80	63.8 ^def^	110	17.4 ^efg^	141
4	DLM	1.20	66.1 ^i^	114	18.5 ^hi^	150
5	DLM	2.10	65.4 ^ghi^	113	20.4 ^k^	165
6	DLM	3.00	65.8 ^hi^	113	20.0 ^k^	162
7	HMTBA	0.40	61.6 ^c^	106	14.7 ^bc^	119
8	HMTBA	0.80	62.9 ^cde^	108	16.4 ^de^	133
9	HMTBA	1.20	64.2 ^efgh^	111	17.3 ^ef^	140
10	HMTBA	2.10	64.1 ^efg^	110	18.4 ^ghi^	150
11	HMTBA	3.00	65.0 ^fghi^	111	19.7 ^jk^	160
12	DLM65	0.40	60.0 ^b^	103	14.0 ^b^	114
13	DLM65	0.80	62.4 ^cd^	107	15.7 ^cd^	128
14	DLM65	1.20	64.7 ^fghi^	111	17.6 ^fgh^	143
15	DLM65	2.10	65.4 ^fghi^	112	18.9 ^ij^	154
16	DLM65	3.00	65.5 ^ghi^	113	20.0 ^k^	163
*p*-value			<0.001		<0.001	

^1 a–k^ Values without a common superscript within a column differ significantly (*p* ≤ 0.05); ^2^ Rel: Relative response of each dietary treatment compared to the basal diet, which was set as 100%.

**Table 5:** Relative bioavailability values (RBV) of DL-2-hydroxy-4-methylthio butanoic acid (HMTBA) and 65% purity diluted DLM (DLM65) relative to DL-Methionine (DLM) for various performance criteria at day 35 determined by multi-exponential regression analysis.

Parameters	Model	Relative Bioavailability Value ^1^		H0:δβ ^3^
		HMTBA	DLM65	
Body weight gain	y = 1440 + 1103 × (1 − e^-(2.41*DLM + 1.40*HMTBA + 1.34*DLM65)^)	58% (48.9–67.7) ^2^	56% (47.2–64.0)	0.608
Feed conversion ratio	y = 1.892 − 0.432 × (1 − e^-(2.00 × DLM + 1.33 × HMTBA + 1.07 × DLM65^)	66% (53.7–78.8) ^2^	54% (42.8–64.2)	0.204
Carcass yield	y = 57.892 + 7.74 × (1 − e^-(2.09 × DLM + 1.32 × HMTBA + 1.22 × DLM65)^)	63% (38.5–87.2) ^2^	58% (33.8–82.7)	0.083
Breast meat yield	y = 12.234 + 8.196 × (1 − e^-(1.22 × DLM + 0.79 × HMTBA + 0.79 × DLM65)^)	65% (52.1–77.3) ^2^	65% (51.3–77.8)	0.255
	**Average relative bioavailability**	**63%**	**58%**	

^1^ RBV determined as ratio of steepness value for HMTBA or DLM65 to DLM according to Littell et al. [15]. ^2^ Confidence intervals at *p* < 0.05 exclude 88%, which is the minimum content of methionine hydroxy analogue in HMTBA and, therefore, RBV are significantly lower. ^3^ Probability of error for testing null hypothesis that asymptotes for the tested three products would differ. *p* > 0.05 indicate common asymptote.

**Table 6:** Compilation of the literature findings and results of the current study on relative bioavailability value (RBV) of DL-2-hydroxy-4-methylthio butanoic acid (HMTBA) and to 65% purity diluted DLM (DLM65) compared to DL-Methionine (DLM).

		Weight Gain		Feed Conversion Ratio		Breast Meat Yield	
Study	Year	DLM65	HMTBA	DLM65	HMTBA	DLM65	HMTBA
1 ^1^	1999	59%	57%	66%	58%		
2 ^2^	2002	60%	68%	57%	67%	69%	64%
3 ^3^	2005	67%	64%	59%	67%		
4 ^3^	2005	69%	63%	79%	73%		
5 ^3^	2005	59%	65%	47%	49%		
Current	2020	56%	58%	54%	66%	65%	65%
Average		62%	63%	60%	63%	67%	65%
**Overall weighted average across all criteria**			**DLM65**	**HMTBA**			
			**62%**	**63%**			

^1^ [13] ^2^ [11] ^3^ [12].
